# Supplementary material for: Genetic Diversity and Association Analysis for Carotenoid Content among Sprouts of Cowpea (Vigna unguiculata L. Walp)
Source: Int J Mol Sci. 2022 Mar 28;23(7):3696. doi: 10.3390/ijms23073696 (PMC8998333; doi:10.3390/ijms23073696)
Supplement: Supplementary file 1 [file ijms-23-03696-s001.zip › Supplementary Figures_Sodedji et al..pdf]

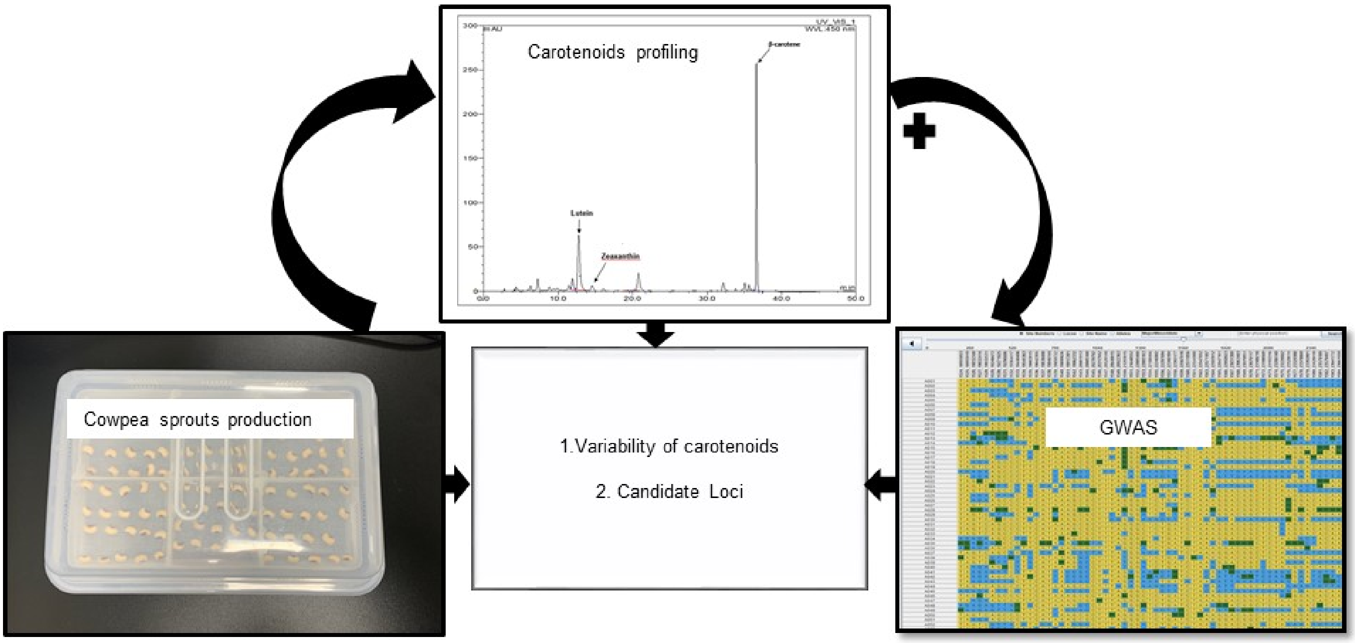


**Figure S1. Schematic diagram showing the approach used in the study**. Briefly, sprouts of 125 cowpea accessions were produced in germination trays, followed by the quantification of the variation for the targeted carotenoids using HPLC, and the genome wide scanning (GWAS) of the associated genetic markers associated in Tassel v5.2.60.

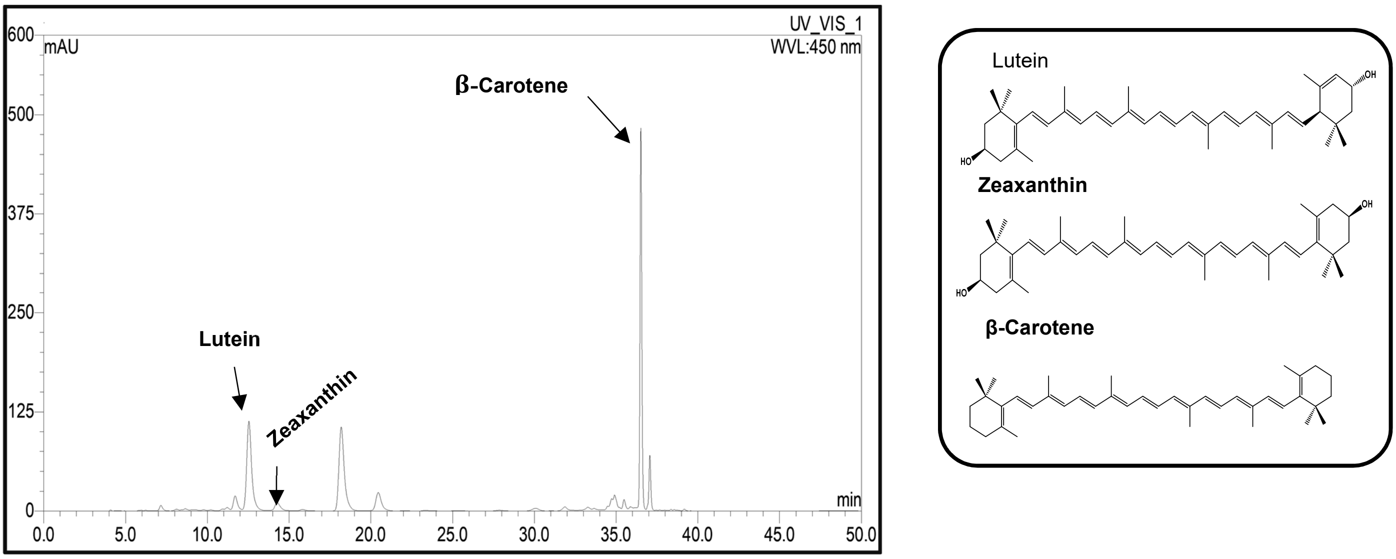


**Figure S2. HPLC chromatograms of the quantified carotenoids**, namely Lutein, zeaxanthin, and β-carotene along with their chemical structures indicated in the box on the left.

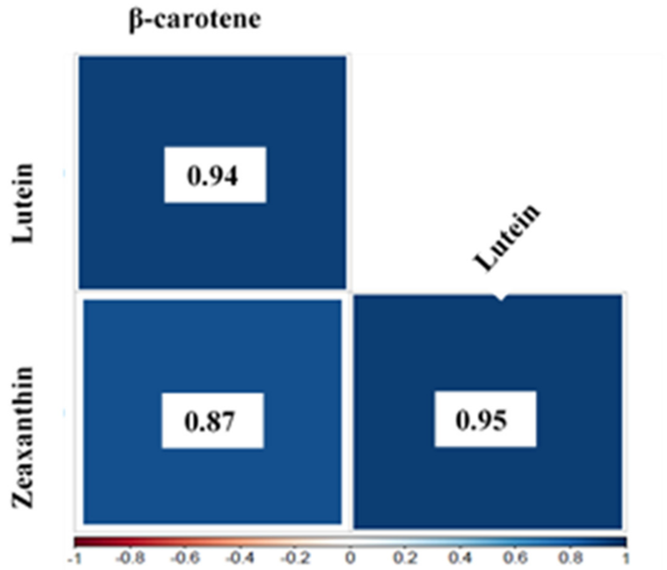


**Figure S3. Correlation analysis among carotenoid compounds.**

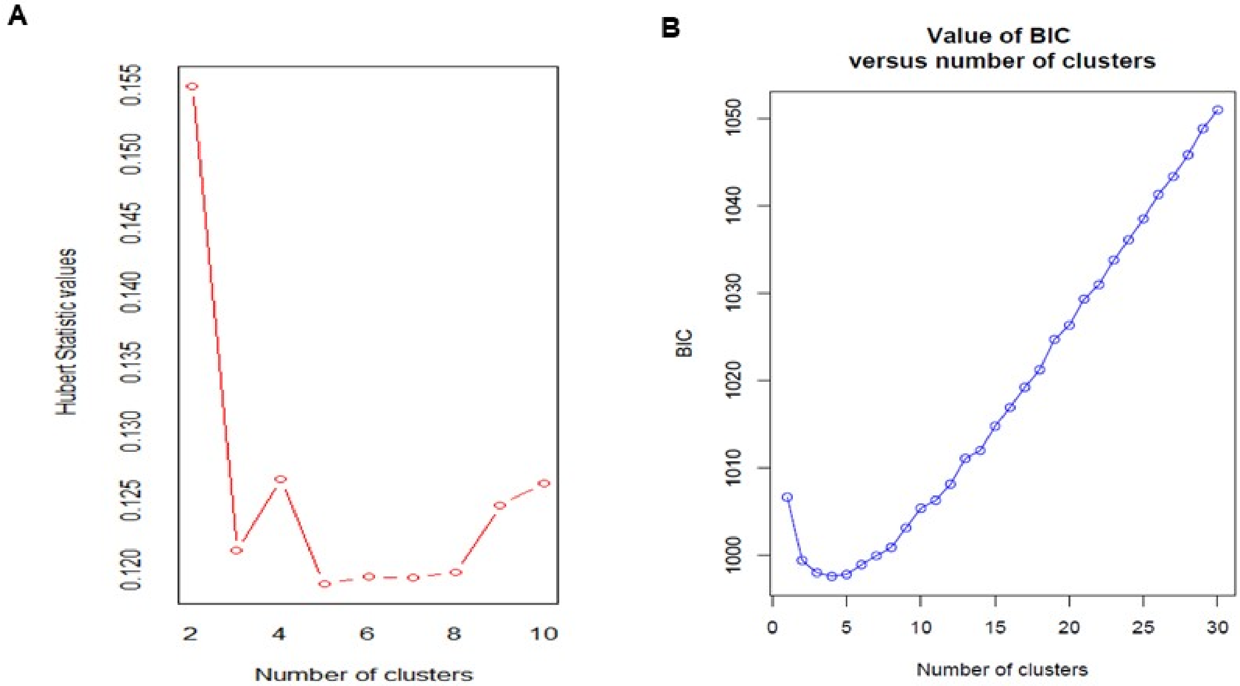


**Figure S4.** Number of clusters in the germplasm **(A)** inferred based on the carotenoid contents with the Neighbor Joining method, whereby the optimal number of cluster corresponds to a knee with significant increase of the value of the Hubert Index; **(B)** inferred in the DAPC approach based on the genetic data; with the graph of the Bayesian Information criterion versus the numbers of cluster showing K=3 as the optimal number of clusters.

A

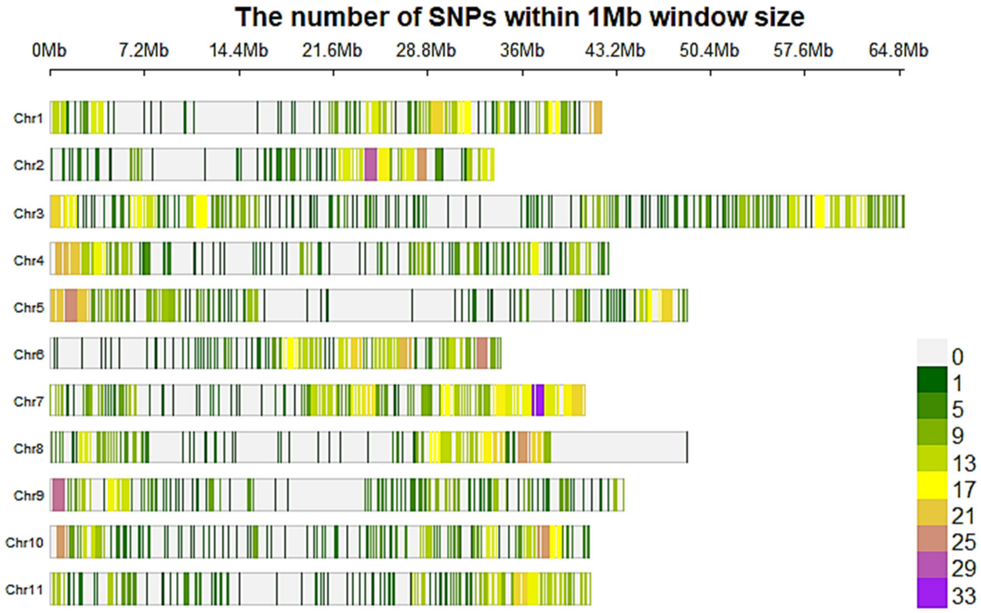


B

| Size | Ho | Hs | Fis | Fst | Gst | Nm |
|---|---|---|---|---|---|---|
| 125 | 0.04 | 0.23 | 0.84 | 0.25 | 0.23 | 5.71 |

**Figure S5**. (**A**) Distribution and densities of the 3120 SNPs across the 11 chromosomes of cowpea and (**B**) genetic diversity indices among the accessions based on Nei method.

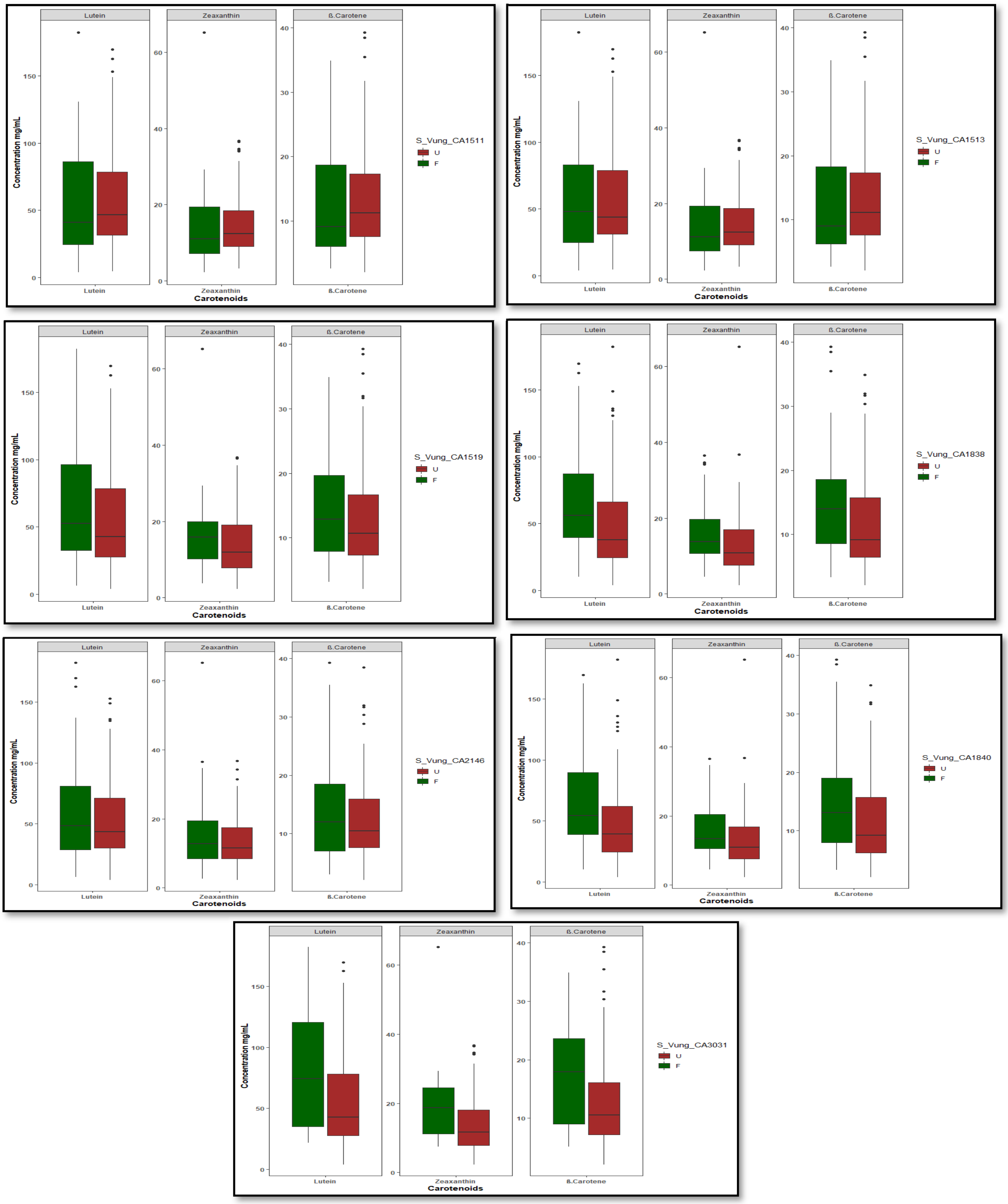


**Figure S6**. Variation in carotenoids contents between groups of cowpea accessions with favorable (F; dark green color) and unfavorable allele (U, Brown color) for 7 signiifcant loci.
